# Isolated Limb Infusion for Limb-Threatening, Unresectable Sarcoma: Past Progress, Current Applications, and Future Directions

**DOI:** 10.3390/jcm12124036

**Published:** 2023-06-13

**Authors:** Danielle K. DePalo, Jonathan S. Zager

**Affiliations:** Department of Cutaneous Oncology, Moffitt Canter, Tampa, FL 33612, USA; danielle.depalo@moffitt.org

**Keywords:** isolated limb infusion, regional chemotherapy, sarcoma, soft tissue sarcoma

## Abstract

Treatment of soft tissue sarcomas (STSs) is complicated by disease heterogeneity. Further, it has not benefitted much from the recent therapeutic advances in other soft tissue malignancies. Surgical resection remains the gold standard in resectable disease, but unresectable, locally advanced STS requires alternative and multimodal approaches. Isolated limb infusion (ILI) provides regional chemotherapy to extremity STS and offers the potential for limb salvage. Despite being in use for nearly 3 decades, there is limited literature on ILI in STS. This review provides an overview of patient eligibility, the procedure, significant publications in this field, and opportunities for further progress.

## 1. Introduction

Soft tissue sarcomas (STSs) are a heterogenous group of malignancies affecting bone and soft tissues [1]. The diversity of these diseases, with over 100 different histological and molecular subtypes of STS, makes identifying effective therapies especially difficult. While there has been a wave of progress in the treatment of cutaneous and other malignancies with the development of immunotherapies and targeted therapies, this has not yet translated to significant improvement of outcomes in STS [2]. The extremities are the most common site of STS origin, with the thigh being the most common site in the extremities [3]. Historically, amputation was considered the standard of care for unresectable STS of the extremity; however, one undeniable advancement for patient quality of life is that amputation is now rarely considered as a first-line treatment since it has not been shown to improve metastatic-free and overall survival [4,5].

While regional chemotherapy, including isolated limb infusion (ILI), was initially developed for the treatment of other malignancies, it has been subsequently applied to the treatment of STS [6,7]. In unresectable STS, recommended treatment options now include primary radiation therapy, chemoradiation, chemotherapy, and regional limb therapy using ILI or isolated limb perfusion (ILP) [8]. Regional limb therapy with ILI or ILP minimizes the systemic effects of chemotherapy through limiting treatment to the affected limb. It should be considered especially in those who would otherwise require amputation as part of definitive surgical management and in those who may not tolerate systemic therapy. In ILP for STS, the National Comprehensive Cancer Network (NCCN) recommends the addition of tumor necrosis factor-alpha (TNF-α) to melphalan or doxorubicin, which does not have Food and Drug Administration approval in the United States. Despite this, ILP is still performed for the treatment of STS in the United States with a debatable impact on efficacy [9,10]. Further, ILI has been shown to be a less invasive and effective alternative to ILP [7]. The aim of this manuscript is to review the considerations for patient selection, provide an overview of the procedure, discuss the available literature, and consider pathways for future investigation of ILI in STS.

## 2. Isolated Limb Infusion Patient Selection and Procedure

Preoperative patient selection should include assessing for sites of distant disease as well as feasibility of performing the regional infusion procedure (ILI or ILP). While ILI is not contraindicated in metastatic STS and may be used as a modality to control limb disease along with multimodal treatments for distant disease as a combination strategy for those with disease outside of the treatment field, it is important to identify where the target disease lies in relation to where the tourniquet would be placed. Very proximal limb lesions may be located proximal to where a tourniquet would lie and, therefore, would not be successfully treated using ILI. Relative contraindications include peripheral vascular disease, decreased kidney function and severe baseline lymphedema. Absolute contraindications are related to inability to undergo general anesthesia and allergy to chemotherapy or heparin products. Additional absolute contraindications include severe peripheral vascular and absent distal extremity pulses in the affected limb as well as barriers prohibiting vascular catheterization, such as vascular stenosis, severe calcification, and prior vascular surgery at the access site disease (especially those patients with synthetic arterial bypass grafts). Prior treatment including ILP or ILI, surgical resection, radiation in the treatment field, and local or systemic therapy are not contraindications.

Melphalan with actinomycin D or doxorubicin monotherapy are recommended in the NCCN guidelines as the chemotherapeutic agents for use in ILI to treat sarcoma [8]. Since the chemotherapeutic dosing is volume-based, preoperative preparation also includes measuring limb volume, either through volumetric displacement or sequential circumferential measurements.

ILI protocols, including our institution’s protocol, have been defined in detail in past literature, and specifics may vary among institutions, but a brief overview of our institution’s procedure is provided here for context [7,11,12,13,14]. ILI differs from ILP in the percutaneous insertion of the catheters, the lack of extracorporeal perfusion, the use of a lower flow and lower pressure circuit, and the shorter duration of the procedure. Percutaneous catheter placement is performed by the interventional radiology department at our institution, with the surgeon first marking the ideal location for catheter tips, approximately 2 cm distal to the tourniquet site and proximal to the lesion(s), using a radiopaque marker (in our institution, a simple paperclip works well). The Seldinger technique and fluoroscopic guidance is used for catheter placement and positioning, and the first dose of heparin is administered at this time. The patient is then transported to the preoperative holding area. The limb is heated prior to infusion with melphalan, ideally to at least 37 °C. Preoperative antibiotics, antiemetics, stress ulcer prophylaxis, and steroids are administered within 30 min prior to the start of the procedure.

**Figure 1:**
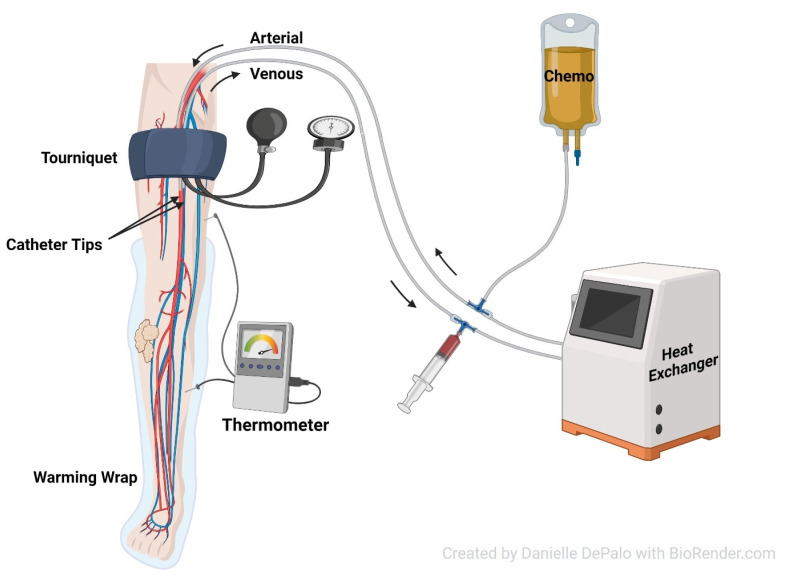
Diagram of isolated limb infusion for soft tissue sarcoma.

In the operating room, the patient is placed in the supine position and placed under general anesthesia. An indwelling urinary catheter with a temperature probe and subcutaneous temperature probes are placed, and the limb is wrapped in a heating device. The patient is connected to the circuit using the previously placed venous and arterial catheters, as seen in Figure 1. The circuit includes a syringe connected to venous outflow for manual circulation, a heat exchanger, and the chemotherapy connected to the arterial inflow. Heparin is administered with a target activated clotting time (ACT) of greater than 400 s, which is monitored throughout the procedure. 

Peripheral arterial pulses are identified using a Doppler ultrasound device and the Doppler is kept in place while the pneumatic tourniquet is inflated. Loss of pulses on the Doppler confirms adequate arterial occlusion and audible flushing of the arterial catheter on the Doppler confirms tip placement distal to the tourniquet. A vasodilator such as papaverine may be administered. Then, the chemotherapy, diluted in heparinized saline, is administered with a pressurized infusion bag over 2–5 min. The circuit is then closed and manually circulated for 30 min using the syringe at a rate of 80–100 mL/L/s. The heater is turned off, and the circuit is opened and flushed with 1 L of isotonic crystalloid until the effluent runs clear. The tourniquet is removed, and ACT is collected and used to dose protamine sulfate for heparin reversal. Catheters are removed once ACT has returned to baseline and direct pressure is applied for a duration dictated according to the catheter diameter.

The lack of extracorporeal perfusion in ILI results in progressive hypoxia and acidosis during the procedure, enhancing the anti-tumor effects of cytotoxic agents [15,16]. This also results in higher limb toxicity following ILI compared to ILP, though ILI has less frequent and severe long-term morbidity and systemic effects, likely due to the lower pressure and lower flow circuit [15,16,17]. Limb toxicity is assessed throughout the hospitalization and at follow-up visits using the Wieberdink grading system [18]. Grade I has no visible changes. Grade II has slight and Grade III has considerable erythema and/or edema. Widespread epidermolysis and/or obvious deep tissue injury with threatened or actual compartment syndrome is seen in Grade IV. Finally, severe tissue damage warranting amputation is seen in Grade V. 

In the postoperative period, the patient is restricted to bedrest and limb elevation overnight and permitted to ambulate the following morning. Hourly neurovascular exams are performed for the first 24 h to identify early signs of compartment syndrome. Immediate mechanical deep vein thrombosis prophylaxis is used in both legs, even in the treated limb, in addition to chemical prophylaxis. On postoperative day 1, monitoring of serum creatinine phosphokinase begins and continues every 12 h until levels peak and return to baseline. CPK greater than 1000 U/L is treated with corticosteroids and gentle fluid resuscitation. 

Following ILI, patients are also monitored for response using direct measurement on clinical examination approximately every 6 weeks and radiographs approximately every 12 weeks. Magnetic resonance imaging is the modality of choice for evaluating extremity STS, but computed tomography or positron emission tomography may also be utilized to evaluate distant disease. Formal response may be assessed using the Response Evaluation Criteria in Solid Tumors version 1.1, as is employed in many of the trials subsequently discussed [19]. Patients who have a mixed or partial response to ILI may be eligible to undergo subsequent ILI procedures. This is an advantageous difference from ILP, in which the procedure may only be performed once.

## 3. Application of Isolated Limb Infusion in Soft Tissue Sarcoma and Available Literature

The NCCN guidelines’ current recommendations for primary treatment of unresectable STS include radiation therapy, chemoradiation, chemotherapy, or regional therapy with ILI or ILP [8]. ILI/ILP is also recommended for consideration in combination with surgery in isolated regional disease with nodal involvement. Despite these recommendations, the published data are limited to relatively small reviews and a single phase II trial that included 2 patients with STS [10,12,13,20,21,22,23,24,25]. Additionally, to our knowledge, there has never been a direct comparison of ILI to systemic, surgical, or other treatment options in unresectable STS. 

One of the earliest works regarding ILI for STS Hegazy et al. in 2007 evaluated the combination of ILI with doxorubicin and external beam radiation (XRT) (total 35 Gy) in 40 patients with unresectable STS of the limb from 2002 to 2005 who were otherwise facing amputation (Table 1) [20]. ILI duration was 15–25 min and general anesthesia was not used. Overall response rate (ORR) was 85%, with 0% complete response (CR), rendering most with resectable disease 3–7 weeks after ILI, for a limb salvage rate of 83% at a median follow-up of 15 months. At median follow-up, 13% had local recurrence and 46% developed distant disease. 

In 2008, Möller et al. published the first review of toxicity data in ILI compared to ILP including sarcoma as well as melanoma [21]. Similar rates of regional toxicity were reported between the 2 modalities, with most being Wieberdink grade II–III and <5% resulting in severe regional toxicity. ILI did have the benefit of having reduced systemic toxicity and the possibility for repeat procedures, if indicated. 

Another early publication on the efficacy of ILI in STS was by Moncrieff et al. in 2008, who reported prospectively collected data on 21 patients from 1994–2007, some of the earliest cases of ILI for STS [22]. Drugs used in the study varied. After 1996, patients received melphalan 5–10 mg/L and actinomycin D 50 to 100 µg/L. From 1994–1996, 1 patient received melphalan plus actinomycin D, 3 patients received mitomycin C with melphalan, and 1 patient received mitomycin C, doxorubicin, and cisplatin. ILI was 20–30 min in duration. Wieberdink grade IV toxicity was seen in 14%, with the remainder experiencing grade II–III toxicity. ORR was 90%, with CR in 57% of patients and a limb salvage rate of 76%. In 14 patients who received ILI in the neoadjuvant setting prior to definitive surgery, ORR was 100% in this cohort with CR in 65% of patients. The other 7 patients underwent ILI for inoperable recurrence or palliation. Procedural factors associated with improved response included lower initial skin temperature with rapid temperature increase and initial PaO_2_ ≥ 194 mmHg.

Local recurrence was seen in 42% of patients with a median local recurrence-free survival (RFS) of 25 months [22]. Local recurrence was significantly reduced, 21%, in the surgical cohort (*p* = 0.013). Improved local recurrence and disease-free survival (DFS) was also associated with CR and the malignant fibrous histiocytoma subtype (now known as undifferentiated pleomorphic sarcoma, UPS), but not the disease stage. At a median follow-up of 28 months, OS was 62% and disease stage was the only predictor of OS. The addition of adjuvant XRT in 48% of patients did not impact OS, DFS, or local recurrence rates.

A phase II single-institution clinical trial published by Brady et al. in 2009 evaluated ILI with melphalan and actinomycin D in unresectable STS and recurrent melanoma of the extremity from 1999–2006 [23]. ORR was 53% at 3 months, with CR in 25% of patients. However, is important to note that only a 20 min circulation time was used, and most significantly, only 2 of the 36 patients in the trial had STS, 1 of whom underwent early amputation for preexisting disease-related pain and was not evaluable for response.

A multi-institution study of ILI with melphalan and actinomycin D in locally advanced STS and nonmelanoma cutaneous malignancies was conducted by Turaga et al. in 2011 [24]. Of the 22 patients, 14 had STS. In those with STS, ORR was 75%, with CR in 17% of patients, resulting in limb preservation in 78% of this cohort. Median progression-free survival (PFS) in the STS cohort was 8.9 months. In all patients, 96% experienced Wieberdink grade III or less toxicity, while 1 patient (4%) had grade IV toxicity.

Beasley et al. sought to compare ILI between the upper and lower extremities, across multiple institutions and malignancies in 2012 [25]. Sarcoma was seen in 5 upper extremity cases (11%) and 16 lower extremity cases (8%). Unfortunately, outcomes specific to the sarcoma cases were not reported. In all cases, greater physiologic derangements were seen in upper extremity ILIs, including base excess (−13.9 vs. −9.1, *p* < 0.001) and pH (7.06 vs. 7.15, *p* < 0.001), though grade 3 or higher toxicity was greater in lower-limb ILIs (7% vs. 24%, *p* = 0.005). Response was only assessed in those that received ILI for melanoma, but there was no difference in CR between the upper- and lower-limb ILIs (28% vs. 32%, *p* = 0.58).

A single-institution review was performed by Vohra et al. in 2013 of 22 patients with locally advanced, limb-threatening sarcoma treated with ILI from 2008–2012 [12]. Similar to the findings of Beasley et al., physiologic derangements were greater in upper-limb ILI and toxicity was more prevalent and more severe in lower-limb ILI [12,25]. CPK peaked later (2 vs. 4.5 days, *p* = 0.02), but not at a significantly higher value in lower-limb compared to upper-limb ILI [12]. ORR was 42% at 3 months, with CR in 24% of cases, and 41% of patients had progressive disease (PD). These response rates did not differ significantly between the upper and lower limb, and they did not correlate with the histologic subtype.

In 2017, Mullinax et al. expanded on the single-institution work of Vohra et al. to include 5 institutions who treated 77 patients with ILI from 1994–2016 for advanced extremity STS, in what remains the largest original study dedicated to ILI in STS, to our knowledge [12,13]. In all cases, melphalan and actinomycin D were circulated for 30 min [13]. The most common subtype was UPS at 44%, followed by synovial sarcoma and leiomyosarcoma (both 6.5%). Physiologic derangements and toxicities were consistent with prior studies [12,13,25]. Unlike Vohra et al., Mullinax et al. also compared upper limbs to lower limbs and observed significantly improved ORR in the lower limb (37% vs. 66%, *p* = 0.03) [12,13]. This did not translate to improved local RFS, distant metastasis-free survival (DMFS), or DSS, but OS was improved in lower- compared to upper-limb ILI (56.6 vs. 27.9 months, *p* = 0.0389) [13].

In all cases, ORR at 3 months was 58%, with CR in 30% [13]. Median follow-up was 20.6 months and median OS was 44.3 months. Response was not associated with severity of toxicity. Response was associated with improved local RFS (16.9 vs. 2.7 months, *p* < 0.0001) and DMFS (not reached (NR) vs. 13.6 months, *p* = 0.02), but not DSS (NR vs. 32.2 months, *p* = 0.2) or OS (44.3 vs. 32.2 months, *p* = 0.9). Ultimately, 22% required amputation for progressive disease at a median of 4.5 months, though responders had a significantly longer time to amputation (NR vs. 12.9 months, *p* = 0.0001).

Additionally, Mullinax et al. conducted a comparison of 71 patients who underwent amputation for locally advanced STS [13]. Progression with distant disease was seen in 48% of cases, median DMFS was 6.4 months, and median OS was not reached at an unspecified follow-up duration. While this suggests that ILI has improved DMFS compared to amputation in this setting, Mullinax et al. did not directly compare the therapies.

A systematic review and meta-analysis was performed by Neuwirth et al. in 2017 on 19 studies evaluating the treatment of locally advanced or marginally resectable STS, including both ILP and ILI [10]. In 1288 patients, 76% had disease of the lower extremity; UPS was the most common subtype, occurring in 21% of patients; and 12% were treated with ILI. Chemotherapeutics used in the limb therapies included 78% melphalan with TNF-α, 10% melphalan with or without actinomycin D, and 12% were other therapies. ILI was compared to ILP with non-TNF-α-based regimens, and ILI had a higher rate of CR (40% vs. 10%, *p* < 0.001) and limb salvage (79% vs. 71%, *p* = 0.03), but similar ORR (72% vs. 73%, *p* = 0.77). Further comparison of ILI with ILP was not reported.

O’Donoghue et al. also expanded on the single-institution work of Vohra et al. in 2017 to include 163 patients who underwent ILI, including 48 with STS from 2007–2016 [12,14]. In all cases, physiologic derangements, CPK trends, and toxicity trends aligned with previously reported data [12,13,14,25]. In the sarcoma cohort, ORR was 49% at 3 months, with CR in 13% of patients [14]. ORR did not differ significantly when comparing upper vs. lower extremities (41% vs. 52%, *p* = 0.5). Similar to Mullinax et al., responders had significantly longer in-field PFS (13.0 vs. 2.7 months, *p* < 0.0001), though this did not translate to improved DMFS (NR vs. NR) or OS (NR vs. 52.8 months, *p* = 0.48) at a median follow-up of 19.3 months [13,14]. The limb salvage rate was 68% [14].

Another single-institution series by Teras et al. in 2019 reported the retrospective outcomes of 10 patients who underwent ILI with melphalan and actinomycin D for unresectable, locally advanced STS from 2014 to 2018 [26]. At 3 months, ORR was 100% with CR in 30% of patients. Median follow-up was 9.5 months and median DMFS was 8.8 months. The limb salvage rate was 80% at a median of 10.1 months. It is important to note that 2 of the 10 patients had benign desmoid fibromatosis, and in 2 cases, ILI was used as adjuvant therapy. 

## 4. Conclusions and Future Directions

While ILI has been integral to the treatment of unresectable extremity STS for nearly 3 decades, there has not been much advancement within the field. Despite a lack of evolution in ILI for STS, recent trends from the National Inpatient Sample suggest that ILI/ILP use for STS has remained stable [9]. Developments that have occurred include relative standardization of the procedure, chemotherapies used, and drug dosing across institutions. Further, we now understand that the ORR for ILI in advanced, unresectable STS is 42–75%, that response rates may be better in lower extremities, that ILI results in limb salvage in 68–79%, and that response to ILI predicts local disease control but not necessarily improved survival [10,12,13,14,24]. 

In melanoma, it is understood that ILI is more effective when used earlier and for a lower burden of disease [27]. Though this association is not well-defined in sarcoma, if anything, we have seen a shift away from the neoadjuvant use of ILI in early publications, despite promising results, to use primarily in the advanced, unresectable, or recurrent setting in more recent publications [10,12,13,20,22,23,24,28]. Perhaps in looking forward, we should look to the past and reconsider the role of neoadjuvant ILI in high-risk and borderline-resectable STS. Additionally, 2 of the patients recently reported by Teras et al. received ILI in the adjuvant setting following resection of disease [26]. Though outcomes specific to those patients were not reported, the use of ILI as adjuvant therapy in resected STS is worth additional consideration. While there are many potential indications for ILI in STS beyond those recommended in oncological guidelines at this time, more evidence is needed before these are implemented into our institution’s standard practice. 

In one of the biggest developments since ILI was first used for STS, a phase II clinical trial (NCT04332874) evaluating the efficacy of ILI in combination with pembrolizumab in locally advanced or metastatic extremity sarcoma is now accruing [29]. A single ILI with melphalan and actinomycin D is performed within 18 days of pembrolizumab initiation, which is continued every 3 weeks thereafter. Interim results of 9 enrolled patients—5 with unresectable or multifocal locoregional disease and 4 with advanced locoregional and distant metastatic disease—indicated that at 6 months, PR was seen in 4 patients and AEs were consistent with the known profiles of each therapy. This trial holds promise as a potential therapeutic regimen, but more so because it represents forward progress in the field of ILI for STS. To our knowledge, this is the first clinical trial conducted dedicated to ILI in STS. Further investigation of ILI in combination therapies and the use of alternative drugs for ILI represent additional avenues for growth. 

## Figures and Tables

**Table 1:** Publications evaluating isolated limb infusion in soft tissue sarcoma, outcomes, and important notes.

Publication and Year	Population	Response, Survival, and Limb Salvage Outcomes	Notes
Hegazy et al. [20] 2007	▪ 40 patients, single institution ▪ 2002–2007 ▪ Unresectable STS, used neoadjuvant ILI and XRT followed by surgery	▪ ORR 85%, 0% CR ▪ Most resectable in 3–7 weeks post-ILI ▪ Median f/u 15 mos. ▪ 83% limb salvage	▪ Short (15–25 min) circulation time
Möller et al. [21] 2008	▪ eview of published rates of toxicity in ILI and ILP used for melanoma and STS	▪ No STS-specific results reported	▪ n/a
Moncrieff et al. [22] 2008	▪ 21 patients, single institution ▪ 1994–2007 ▪ ILI in 14 as neoadjuvant or 7 as unresectable/palliative	▪ In all, 3-month ORR 90%, CR in 57% ▪ In 14 neoadjuvant, ORR 100%, CR 65% ▪ Median f/u 28 mos. ▪ In all, local RFS 25 mos. Additionally, OS 62 mos. ▪ In all, 76% limb salvage	▪ Alternative drug regimens used in earliest cases ▪ Variable circulation time (20–30 min) ▪ Outcomes not reported specifically for 7 unresectable/palliative
Brady et al. [23] 2009	▪ 2 patients, phase II trial, single institution ▪ 1999–2006 ▪ Unresectable STS	▪ No STS-specific results reported	▪ 34 additional patients had recurrent melanoma; reported outcomes included all indications and only 1 case with STS was evaluable for response ▪ Shorter (20 min) circulation time
Turaga et al. [24] 2011	▪ 14 patients, multiple institutions ▪ 2004–2009 ▪ Locally advanced STS	▪ 3-month ORR 75%, CR 17% ▪ PFS 8.9 mos. ▪ 78% limb salvage	▪ 8 additional patients who underwent ILI for other indications, only STS-specific results reported here
Beasley et al. [25] 2012	▪ Multi-institution ▪ ILI for multiple malignancies ▪ STS in 5 UE cases and 16 LE cases	▪ No STS-specific results reported	▪ n/a
Vohra et al. [12] 2013	▪ 22 patients, single institution ▪ 2008–2012 ▪ Locally advanced, limb-threatening STS	▪ 3-month ORR 42%, CR 24% ▪ ORR same between UE and LE and same between histologic subtypes	▪ n/a
Mullinax et al. [13] 2017	▪ 77 patients, 5 institutions ▪ 1994–2016 ▪ Locally advanced STS	▪ 3-month ORR 58%, CR 30% ▪ ORR and OS improved in LE compared to UE ▪ Median f/u 20.6 mos. ▪ Responders had improved local RFS and DMFS, not DSS or OS ▪ 78% limb salvage	▪ Looked at 71 patients who had amputation as well, DMFS appears to be improved with ILI but not directly compared to amputation
Neuwirth et al. [10] 2017	▪ Systematic review and meta-analysis of 19 studies ▪ 1288 patients, 12% ILI	▪ ORR 72%, CR 40% ▪ 79% limb salvage ▪ CR and limb salvage were significantly higher than ILP with non-TNF-α-based regimen	▪ Only presented data on ILI compared to ILP with non-TNF-α-based regimen
O’Donoghue et al. [14] 2017	▪ 48 patients, single institution ▪ 2007–2016 ▪ Unresectable, limb-threatening STS	▪ 3-month ORR 49%, CR 13% ▪ ORR same between UE and LE ▪ Median f/u 19.3 mos. ▪ Responders had improved in-field PFS, not DMFS or OS ▪ 68% limb salvage	▪ 115 additional patients underwent ILI for other indications, only STS-specific results reported here
Teras et al. [26] 2019	▪ 10 patients, single institution ▪ 2014–2018 ▪ Unresectable, locally advanced STS	▪ 3-month ORR 100%, CR 30% ▪ Median f/u 9.5 mos. ▪ DMFS 8.8 mos. ▪ 80% limb salvage	▪ 2 of 10 patients had benign desmoid fibromatosis and 2 of 10 patients received ILI as adjuvant therapy; outcomes were not reported separately

Abbreviations: STS, soft tissue sarcoma; ILI, isolated limb infusion; XRT, external beam radiation therapy; ORR, overall response rate; CR, complete response; f/u, follow-up; ILP, isolated limb perfusion; n/a, not applicable; RFS, recurrence-free survival; OS, overall survival; PFS, progression-free survival; UE, upper extremity; LE, lower extremity; DMFS, distant metastasis-free survival; DSS, disease-specific survival; TNF-α, tumor necrosis factor-alpha.

## Data Availability

Not applicable.
